# Effectiveness of community based safe motherhood promoters in improving the utilization of obstetric care. The case of Mtwara Rural District in Tanzania

**DOI:** 10.1186/1471-2393-10-14

**Published:** 2010-04-01

**Authors:** Declare Mushi, Rose Mpembeni, Albrecht Jahn

**Affiliations:** 1 Department of Community Health, Tumaini University-Kilimanjaro Christian Medical Centre, P.O. Box, 2240, Moshi, Kilimanjaro, Tanzania; 2 School of Public Health and Social Sciences, Muhimbili University of Health and Allied Sciences, P.O Box 65015, Dar es Salaam, Tanzania; 3 Institute of Public Health, Ruprecht-Karls-University, Im Neuenheimer Feld 324, 69120 Heidelberg, Germany

## Abstract

**Background:**

In Tanzania, maternal mortality ratio remains unacceptably high at 578/100,000 live births. Despite a high coverage of antenatal care (96%), only 44% of deliveries take place within the formal health services. Still, "*Ensure skilled attendant at birth*" is acknowledged as one of the most effective interventions to reduce maternal deaths. Exploring the potential of community-based interventions in increasing the utilization of obstetric care, the study aimed at developing, testing and assessing a community-based safe motherhood intervention in Mtwara rural District of Tanzania.

**Method:**

This community-based intervention was designed as a pre-post comparison study, covering 4 villages with a total population of 8300. Intervention activities were implemented by 50 trained safe motherhood promoters (SMPs). Their tasks focused on promoting early and complete antenatal care visits and delivery with a skilled attendant. Data on all 512 deliveries taking place from October 2004 to November 2006 were collected by the SMPs and cross-checked with health service records. In addition 242 respondents were interviewed with respect to knowledge on safe motherhood issues and their perception of the SMP's performance. Skilled delivery attendance was our primary outcome; secondary outcomes included antenatal care attendance and knowledge on Safe Motherhood issues.

**Results:**

Deliveries with skilled attendant significantly increased from 34.1% to 51.4% (ρ < 0.05). Early ANC booking (4 to 16 weeks) rose significantly from 18.7% at baseline to 37.7% in 2005 and 56.9% (ρ < 0.001) at final assessment. After two years 44 (88%) of the SMPs were still active, 79% of pregnant women were visited. Further benefits included the enhancement of male involvement in safe motherhood issues.

**Conclusion:**

The study has demonstrated the effectiveness of community-based safe motherhood intervention in promoting the utilization of obstetric care and a skilled attendant at delivery. This improvement is attributed to the SMPs' home visits and the close collaboration with existing community structures as well as health services.

## Background

According to the recent review of the Millennium Development Goals (MDG), little progress has been made with respect to MDG 5 on improving maternal health [1]. Skilled delivery care is considered a crucial function within the health care system for saving the lives of mothers and newborns and represents an important indicator for monitoring MDG 5 [2,3]. In Tanzania, pregnancy-related mortality remains unacceptably high with estimated maternal mortality ratio of 578/100,000 and a neonatal mortality rate of 32/1000 live births [4]. Maternal deaths account for about 27% of all deaths in women aged 15-49 [5-7]. Strikingly, despite high ANC attendance (96%), free maternal health care services and an impressive health infrastructure utilization of skilled care at delivery has been decreasing with time from 53% in the late 1980's to 44% in 1999 and rising slightly to 47% in 2005. This shows that more than half of all deliveries (53%) are taking place at home with unskilled attendants [4,8]. While in Tanzania 72% of the population are residing within 5 km and 90% within 10 km of a health facility which provides delivery care, physical accessibility of health facilities does not automatically increase utilization of professional delivery care. This is especially true in rural communities where a mix of socio-cultural, personal, economic and health service factors stand as main barriers [9-12].

Against this background, the relative failure of purely service-based interventions has led to a renewed interest in community-based approaches to address maternal, new-born and child health [13]. In poor resource settings, community-level interventions are potentially effective ways to address the problem at its roots, as decisions to seek and access health care is very much influenced by socio-cultural environment [14,15]. Community-based safe motherhood interventions, if appropriately designed, can effectively address social-economic and cultural barriers at family and community level and can work to improve women's health *prior, during and after *pregnancy [9,16,17]. Training of *change agents *who are commonly known as community volunteers or peers [9,14,18-20] is an important element in most community based health interventions. Their tasks and activities included specific maternal health activities such as follow-up of pregnant women, providing education on danger signs, risk factors, and birth preparedness plans, counselling, to facilitate referrals and advocacy to influence or demand better obstetric care or health services [21-23]. In close collaboration with villagers, their representatives, and health service providers in Mtwara Region, Tanzania, a community-based intervention package for Safe Motherhood was developed and evaluated. This package relied heavily on the involvement of community volunteers, called Safe Motherhood Promoters (SMPs). This paper describes and analyses the process and the effectiveness of this intervention with respect to service utilization, acceptability and community perception. Coverage of skilled delivery attendance, a key indicator for the Millennium Development Goal 5 (Reduce maternal mortality) [24] was used as primary study outcome.

## Methods

### Description of the Study Setting

The study was carried out in the Mtwara Rural District in south-east region of Tanzania from June 2004 to November 2006. Mtwara is one of the least developed regions in country and the majority of its population are poor subsistence farmers earning below 1 US$ per day and hence living under the poverty line. The dominant ethnic group is Makonde. Others are Yao and Makua. 95% of the populations are Muslim. About 40% of the population do not have primary education [4]. In 2005, maternal mortality ratio in the district was estimated at 600 per 100,000 live births and infant mortality is high at 136 per 1000 live births. Mtwara Regional Hospital serves as the first referral level for obstetric emergencies for six districts in the region including Mtwara rural district [25]. In 2006, only 36% of deliveries occur in health institutions [26].

### The Study Site and Population

The study took place in four villages Mahurunga, Kitunguli, Kihimika and Tangazo, with a total population of 8300. All deliveries which occurred between October 2004 and November 2006 (N = 512) were included in the study. The four villages are located in Mahurunga Ward in Kitaya Division. The area is served by one health centre and a dispensary which both provided maternal and child health services including delivery care. The two facilities were staffed by two clinical officers and three skilled midwives. According to the Tanzania health system, these lower level health facilities provide only normal delivery care. In Tanzania, midwives, staff nurses and public health nurses with midwifery skills and mother and child health aide (MCHA) are allowed to perform normal deliveries. Thus, women with risk factors and those who develop obstetric complication are to be referred to Mtwara Regional Hospital, which is 50 km away and the closest place providing comprehensive emergency obstetric care services.

Besides general public awareness education through media, no community-based interventions have previously been carried out in the study area to promote utilization of skilled attendants at delivery.

### The study design and a brief description of intervention

The study was designed as a community-based intervention study, using a pre-post comparison of the same group; which was implemented in three phases: situational analysis June 2004; intervention October 2004 to October 2006 and the post-intervention assessment in November 2006. The intervention package comprised of two main components (a) training of Safe Motherhood Promoters (these were trained specifically for this intervention study) and (b) education and awareness on maternal health aspects. As specified in more detail below, the study comprised a quantitative approach with respect to the pre-defined primary and secondary study outcomes. Responses to selected open-ended questions were also analyzed qualitatively.

#### Community entry and baseline survey

After consultation with the village governments, several meetings were held in each of the four villages. The initial meeting aimed at informing community leaders and other stakeholders and discussing the objectives and the implementation plan with them. After reaching a consensus, the baseline survey was carried out between June and July 2004. This survey exercise was used as initial stage of community entry and relationship building and to prepare the community for the intervention. After the situational analysis exercise, preliminary results and the intervention package were presented and discussed in a second community meeting which involved village government and political leaders, religious and opinion leaders, village health workers, school teachers, health providers Traditional Birth Attendants (TBAs) and traditional healers. Their inputs and recommendations were incorporated in the final intervention package after being reviewed by district health team. This consultation process was continued with further village meetings throughout the study.

### Intervention components

The key elements of the intervention were:

a. Training of safe motherhood promoters, using participatory adult learning methods.

b. To conduct home visits, to educate pregnant women and their husbands and key community members about danger signs and pregnancy complications.

c. Home visits to pregnant mothers to inform on early and complete ANC visits and the importance of a birth preparedness plan

d. Follow-up of pregnant mothers to encourage delivery with a skilled attendant and data recording.

e. Raising awareness on maternal health issues through community meetings and video show

Criteria for the selection of SMPs were established in public meetings and included (1) she/he must be a married person, (2) able to read write, (3) accepted by community, and able to educate others. However, four TBAs and two community representatives who did not know how read and write were selected due to their popularity and acceptance in the community. A total of 50 SMPs (28 females and 22 males) were selected with an average of 10-12 SMPs per village (3-5 from each sub-village). SMPs varied in age from their mid-twenties to late early sixties. The age range was 23-64 years, while the median age was 37 years old. SMPs were drawn from community groups including village government, religious institutions, opinion leaders, community health workers (CHWs), traditional birth attendants (TBA), and village health committees. These SMP's were trained in their villages for 12 days, using a participatory adult learning methods [27,28]. The topics covered conception and pregnancy, complications and danger signs, risk behaviour, birth preparedness, rationale and content of antenatal care as well as the importance of skilled delivery care. The key messages were derived from the Tanzanian National Reproductive Health and Communication Strategy and Safe Motherhood Initiatives Messages [29].

#### Post-training activities

A post training feedback meeting with community leaders and SMPs was held to develop a plan of action on how to implement the intervention activities. The tasks of SMPs that were agreed upon included:

a. To conduct home visits, to educate pregnant women and their husbands and community key members about danger signs, delivery complication and prevention of HIV/AIDS transmission from mother to child.

b. To motivate women and their families to book early for ANC and to have birth plans.

c. To promote and facilitate women to deliver with skilled attendants.

d. To conduct monthly meetings and data recording.

Other post-training activities by SMPs were community awareness activities through educational meetings, supported by video shows and other media outlets.

#### Organizational and implementation structure

Experienced local health officers who participated in the baseline and the training of SMPs coordinated the intervention activities which were also integrated within the existing primary health care (PHC) activities. Each SMP were assigned a maximum of 12 households to care for including data collection and reporting on a monthly basis. The SMP team worked under the Village Health Committee.

#### Follow-up and monitoring activities

Four experienced research assistants in community health were recruited and trained on data collection and ethical issues. All the study tools were translated into Kiswahili and piloted. Necessary changes ware made accordingly. The main researcher conducted field visits and supportive supervision to both SMPs and local coordinators. Problems identified and lessons learned were discussed in a joint meeting with the SMPs, village leaders and the Mother and Child Health (MCH) team.

### Data collection

#### Quantitative data on place of delivery, delivery attendant and use of antenatal care services

Data on all deliveries (N = 512) in the 4 villages were collected by SMPs during home visits, supported and supervised by the research assistants and the study coordinator, for a period of three years (October 2004- to November 2006). The data included information on maternal age and parity, the attendance at ANC clinics, risk factors as identified by the health providers, referral status, place of delivery and the person assisting during delivery. These data sets were crosschecked with health facility records from the study villages, which recorded 459 deliveries (including home deliveries by asking women and through TBAs records) during the study period; fifty-three deliveries less than the number reported by SMP. All of these 53 additional deliveries were followed up and confirmed by the research assistants. Thus, data collected by SMPs were more complete because they lived in the community and each SMP had no more than 12 pregnant women to follow-up per year.

#### Quantitative and qualitative data on safe motherhood knowledge and perception of the intervention

These data were derived from interviewing a random sample of residents of the four villages including female (pregnant, nursing mothers, and mothers) as well as male (husbands of the same) aged 18 years or above. The composition of all households was established in the baseline survey. From this list we randomly selected 238 respondents prior to the intervention and 242 in post-intervention to participate in semi-structure individual interviews (SSIs). SSIs were conducted by the main researcher and two trained research assistants.

The SSI tool consisted of closed and open-ended questions. We collected information on demographic characteristics and on knowledge on referral issues such as risk factors, danger signs, barriers for referrals, and choice of place of delivery in case of non-compliance. We also asked questions on the weaknesses, strengths and the general perceived benefit of the intervention. The interview data were analyzed quantitatively. In addition, responses to open-ended questions on perceptions were analyzed qualitatively with key statements being presented in the results.

In the final assessment in 2006, the 242 respondents included 153 mothers and 69 husbands of those women, as well as 20 women who were pregnant during the finals assessment. The age range for respondents was between 19 and 53 years while the median age was 29 years.

After data collection, a one day community feedback meeting which involved sixty community leaders (23 females and 37 males) from all villages was held. The feedback meetings involved a ward executive officer, village leaders, religious and political leaders, SMPs, school teacher and health providers. A brief report about preliminary findings was presented and main issues emerged from our findings were discussed in four village-based groups and in a large group. The discussion also helped to clarify some of the issues raised during interviews and was used to validate these findings.

### Data analysis

Data from the 512 delivery records, 238 individual interviews (SSI) at pre-intervention and the 242 SSIs at post intervention were entered into a computer program using EpiInfo 6 data entry program. Data cleaning and analysis was done using the same programme. In addition to the primary study outcome which was the coverage of skilled delivery attendance, we also analyzed secondary outcomes which were ANC attendance and knowledge on Safe Motherhood issues. We also performed a process evaluation through assessing the acceptability and appreciation of the SMPs, the coverage of home visits, and the drop-out rates of SMPs.

### Ethical issues

This study was part of a larger study on maternal and perinatal health in Mtwara Region. Ethical approval was granted by the Muhimbili University of Health and Allied Sciences Ethical Committee. Official permission was obtained from the Mtwara Regional Executive Officer, the Mtwara Rural District Administrative Officer, the Mahurunga Ward Executive Officer and all village chairpersons from the study area. Before the interview participant were assured of confidentiality and the written consent was obtained. As part of the ethical obligations preliminary findings were presented and discussed in the community feedback meetings which involved all sub-village and village leaders, religious and opinion leaders, health providers and the safe motherhood promoters.

## Results

### Intervention impact on place of delivery and skilled attendance

With respect to our primary outcome, the results show a significant improvement in the utilization of a skilled attendance with variation across the villages. Deliveries with skilled attendants significantly increased from 34.1% at baseline to 51.4% in 2006 (ρ < 0.05), against a background of almost a static situation at regional level which was 36% in 2004 at baseline and at post-intervention in 2006 [30]. The proportion of women who were assisted by TBAs decreased from 35.7% to 29.9% while those assisted with relatives went down from 30.2% to 17.3% (Table 1). As a new development, skilled health workers started to assist in home deliveries, leading to an increase in skilled attendance for women who delivered at home.

**Table 1:** Place of delivery and type of attendant

Place of delivery	Mahurunga 2005-2006* (n = 136)	Kitunguli 2005-2006* (n = 139)	Kihimika 2005-2006* (n = 99)	Tangazo 2005-2006* (n = 138)	Total 2005-2006* (n = 512)	Baseline 2004** (n = 258)
Institutional deliveries	65 (47.8%)	63 (45.3%)	34 (34.3%)	93 (67.4%)	**255 (49.8%)**	**86 (33.3%)**

Home Deliveries with a skilled attendant	2 (1.5%)	4 (2.9%)	0	2 (1.4%)	**8 (1.6%)**	**2 (0.8%)**

*Total delivery with skilled attendant*	*67(49.3%)*	*67 (48.2%)*	*34 (34.3%)*	*95 (69%)*	***263 (51.4%)***	***88 (34.1%)***

Home delivery with TBA	43 (31.5%)	46 (33.1)	41 (41.4%)	23 (16.7%)	**153 (29.9%)**	**92 (35.7%)**

Home delivery With Relatives	23 (17.0%)	26 (18.7%)	22 (22.2%)	18 (13.1%)	**89 (17.3%)**	**78 (30.2%)**

Delivery on the way to health facility	3 (2.2%)	0	2 (2.0%)	2 (1.4%)	**7 (1.4%)**	**-**

* 2-years period

** 1-year period

### Impact on the Antenatal care attendance

Since utilization of obstetric care is very much linked to ANC visits, promoting early ANC booking and the recommended number of ANC visits was one of the important activities of SMPs. In the study area, the proportion of women who attended ANC at least once during pregnancy was high (94%).

The primigravida mothers who booked ANC early, between 4 to 16 weeks, increased significantly (ρ < 0.01) from 18.7% (12 out of 64) at baseline in November 2004, to 56.9% (41 out of 72) at final assessment in November 2006. The proportion of primigravida women who booked late for ANC, at 20 weeks and above, significantly dropped from 60.9% (39 out of 64) in 2004 to only 16.7% (12 out of 72) in 2006 (p < 0.01). The proportion primigravida of women who had four or more ANC visits increased from 42.2% (27 out of 64) at baseline to 51.3% (37 out of 72) at post-intervention, but this difference was not significant.

### Community knowledge and perception of the safe motherhood issues

Table 2 provides the socio-demographic background of the pre- and post-intervention interviewees asked about their knowledge and perceptions, and thus a proxy for the general situation in the study area. Of all the 242 respondents 62.8% were married, 28.4% were single (separated or divorced) and 7.4% had never married. The age range for respondents was 19 to 53 years with the median age of 29 years. The majority of women (60%) married for first time at mean age of 18 years and became pregnant soon after marriage. Education attainment was generally low. Nearly half (41.3%) had never been to school. Transport to Mtwara town is difficult and the major modes of transport are walking and bicycle.

**Table 2:** Socio-Demographic characteristic of the interviewees

Characteristics	Baseline		Post intervention	
	**Number (n = 238)**	**%**	**Number (n = 242)**	**%**

***Gender***				
Female	138	58%	173	71.5
Male	100	42%	69	28.5
***Age***				
16-19	16	6.7	20	8.3
20-24	71	29.8	68	28.1
25-34	89	37.4	95	41.3
35-49	51	21.4	55	23.9
50+	11	4.6	4	1.7
**Marital Status**				
Married	159	66.8	152	62.8
Separated/	59	24.8	50	20.6
Divorced	12	5.04	19	7.8
Never married	8	3.4	18	7.4
**Religion**				
Moslems	225	94.5	231	95.4
Christians	13	5.5	11	4.6
**Ethnicity**				
Makonde	219	92.1	220	90.9
Others (Yao & Makua)	19	7.9	21	9.1
**Education**				
Never been to School	**93**	**39.1**	**100**	**41.3**
Female	69	29	82	33.9
Male	24	10	18	7.4
Complete Primary Education	**85**	**35.7**	**92**	**38**
Female	53	22.3	58	24
Male	32	13.4	34	14.04
Never complete Pr/Education	**46**	**19.3**	**38**	**15.7**
Female	30	12.6	28	11.8
Male	16	6.7	10	4.1
Secondary	**14**	**5.9**	**9**	**3.7**
Female	5	2.1	4	1.7
Male	9	3.9	5	2.06

Respondents were considered knowledgeable if they were able to mention at least 3 safe motherhood items in each specific area (table 3). At post-intervention in 2006, 128 (52.9%) of respondents were able to mention at least three pregnancy risk factors compared to 110 (46.2%) at baseline in 2004. Thus, an increase in knowledge was observed, but did not reach statistical significance (p > 0.05). The proportion of respondents who were able to spontaneously mention three danger signs during delivery increased slightly from 52.9% at baseline to 56.2% at post-intervention. The percentage of respondents who were able to cite at least three practices that contributes to delay in seeking obstetric care went up from 46.6% at base line to 55.9% at post-intervention. Number of respondents who believed that pregnancy complications are not due to non-observance of traditions increased from 28.7% at baseline to 47.1% at post-intervention in 2006.

**Table 3:** Knowledge on maternal health aspects

	Pre-Intervention (June 2004)			Post-Intervention (Nov, 2006)		
**Proportion of Respondents**	**Female n = 138**	**Male n = 100**	**Total n = 238**	**Female n = 173**	**Male n = 69**	**Total n = 242**

Able to mention at least three risks practices during pregnancy	52 (47.3)	58 (58%)	**110 (46.2%)**	89 (51.4%)	39 (55%)	**128 (52.9%)**

Able to identify three danger signs during pregnancy	72 (52.3%)	54 (54%)	**126 (52.9%)**	94 (54.3%)	42 (60.9%)	**136 (56.2%)**

Able to mention three complications during delivery	66 (47.8%)	41 (41%)	**107 (44.9%)**	92 (53.2%)	36 (52.2)	**128 (52.9%)**

Able to identify at least 3 practices that contributes to delay in seeking care	65 (47.1%)	52 (52%)	**117 (46.6%)**	95 (55.2%)	40 (58%)	**135 (55.9%)**

Respondents who do not believe pregnant complications are due to non-observance of tradition	32 (23.2%)	36 (36%)	**68 (28.7%)**	74 (42.8%)	40 (58%)	**114 (47.1%)**

Sixty five percent of respondents said their main sources of information about pregnancy during the intervention period were SMPs compared to 38.5% who recalled community health workers (CHWs) at baseline. The proportion of respondents who mentioned TBAs as a source of maternal health information increased from 16% at baseline to 27% at post-intervention.

### Performance of safe motherhood promoters and community acceptance

Of the 50 SMPs who were trained 44 (88%) remained and continued to volunteer throughout the study period and only six SMPs dropped out. Thirty six (94%) of SMPs said the training was useful to themselves, their work and to the community they serve. Out of the 38 interviewed SMPs, 35 (92.1%) said they were "very much accepted" by pregnant women, community members and health providers. Only three (7.9%) said they were not well received.

Out of 512 of women who gave birth during the study 404 (79.7%) said they were visited during pregnancy. About half (47.1%) of women were visited at least twice during pregnancy and 34.7% was visited and 85% of the community members were positive about the role of SMPs. Only 17.4% said they had not been visited. Health providers and community leaders were also found to have a positive attitude towards SMPs. SMPs reported that they were very much accepted by the midwives in the health facilities and that they were free to meet them in case of any question.

### Acceptance of Safe Motherhood Promoters

In general, the majority of respondents were positive about the role of SMPs. In the 242 post-intervention interviews, 52.4% of women and 48.5% men were highly impressed by home visiting and the follow-up of pregnant women: *"When SMPs visit us at home we feel good because we have enough time to discuss and to ask questions*" (a nursing mother). When respondents were asked to give their opinions about the performance of SMPs, they expressed their views with phrases such as "*they have done their best*", "*they are committed"*, "*they like their job*" "*although they are not paid they are serious in follow-up*". Only 12.6% of women saw SMPs to be incompetent compared to men (32%). Those who said SMPs were not competent cited the short two weeks training, their advice being limited to safe motherhood and that some of the SMPs were illiterate and thus they couldn't teach.

### Impact of intervention: Expected and unexpected outcome

Some of the parameters to assess the impact of the intervention on the community health system were: links to existing structures, number of active SMPs, involvement of key partners, and coverage of maternity care. Some of the observed positive effects during the course of the study as expressed by community leaders, health providers, SMPs and villagers during monitoring and the in final assessment were the following:

▪ Improved involvement of community leaders at sub-village level in promoting safe motherhood, by supporting and allowing SMPs to present their reports during the village quarterly meetings. Also the study has motivated men in the community to be interested in maternity issues.

▪ Increased involvement of religious leaders in community health activities. A ward secretary commented "*Because they are part of SMPs team, we have seen some of them conducting home visits and heard them promoting and encouraging women to deliver with skilled attendants during worship services"*.

▪ The intervention has built linkages between health providers, SMPs and pregnant women and TBAs. TBAs who were part of SMPs have become active promoters of skilled attendant at delivery and some have changed from delivery care provider to educators and counsellors and referral advisors. Furthermore being part of the team some TBAs' became reluctance to perform home deliveries on their own, particularly in women with specific risk factors.

## Discussion

The study showed three main results. Firstly, community-based interventions which promote access to maternal health services and obstetric care through community volunteers are feasible and effective. Secondly, close collaboration with existing community structures as well as health services is crucial. Thirdly, community-based interventions can have a wider positive impact, such as male involvement in safe motherhood and better collaboration between health services and community.

Deliveries with skilled attendants increased remarkably during the intervention period. As the overall district data remained static, the increase in skilled delivery in our study is most likely due to the SMP intervention and its emphasis on community involvement. This included a continuous dialogue and consultation process, as well as strong link between the community and health service providers through village-based structures such as the village health committee. The study also demonstrates the importance of engaging directly with the community in general and pregnant women in particular through community outreach activities and home visits of pregnant women and their families. While observing a non-significant trend towards improved knowledge, it appears that the personal contact with SMPs during home-visits was the most important factor for improved ANC visits and utilization of a skilled attendant at delivery. Our analysis shows that more than three quarters of pregnant women were visited by SMPs during pregnancy. There was a positive response, because most women accepted the advice of SMPs. Beyond provision of health information, communities have to be organized, mobilized and engaged to implement specific actions that can effect behaviour changes through systematic follow-up that take advantage of social networking mechanism [31]. This finding is in line with the theory of health seeking behaviour which states the decisions to seek care is very much influenced by the social-environment in which one lives [14,15].

It has been argued that within the health care system, community volunteers can be part of primary health care system. The use of community health volunteers as agents of health promotion has been a classical approach in community health and other development programs [21,23,32]. In line with findings from Sierra Leone, Nigeria and Bolivia [33-36], our study shows the potential of community change agents - in our case SMPs - to increase utilization of obstetric care and other maternal health services such as early ANC booking and increased number of ANC visits.

Acceptance of volunteers by the community is important for program success [36]. In general, the majority of respondents, including health providers, did accept and were positive about SMPs and their role in the community. Contrary to the traditional approaches where most safe motherhood interventions uses women as change agents, in this study the inclusion of men (almost 50%) and religious leaders in the SMPs' team made the intervention valued and acceptable. Only a few respondents expressed a feeling that SMPs' training was too short for them to be competent.

Despite being unpaid and with low educational background, the majority of SMPs actively performed their roles as agreed with only six drop-outs out of fifty who were trained. Nearly eighty percent of respondents visited were satisfied with the education and information, education and communication (IEC) material given. SMPs were also accepted by health providers and they felt proud when realizing that their work was valued and supported by professional health workers. Sometimes SMPs would meet with midwives to ask questions and to discuss progress and challenges. This was possible because from the beginning midwives were involved in all stages and in the main activities of the intervention. Midwives also supported SMPs because they found them to be helpful in their community outreach activities. At family and community level, SMPs were involved in advising and encouraging family members and women with detected danger signs to utilize skilled attendants at delivery. Pregnant women were identified by SMPs in two ways; during home visits and through a list provided by the midwives. The two strategies resulted in a high number of women who reported to be advised by SMPs to the first level of care for delivery.

Engendering of safe motherhood programs and strategies is another important aspect. Traditionally, interventions have tried to involve individual husbands/partners to support wives to utilize maternal health services [37,38]. In our study we have been able to demonstrate that men can be organized as a group of change agents that work hand in hand with other community structures in promoting safe motherhood. Half of the SMPs team was men. Since, in traditional societies, men are respected, heard and they make or influence most decisions, the involvement of men in the SMPs team was an important element that contributed to the effectiveness of the intervention. Involving men in maternity care is essential and health planners should be trained to view men as "partners and key players" and not as "barriers". A positive view of men will increase chances of incorporating them in the process of change.

Attendance at ANC in Mtwara is very high and close to the national average of 96% [4,39]. An increased proportion of women attending ANC early in pregnancy and increased number of visits per pregnancy as a result of SMPs promotion activities, reflects accessibility in terms of close proximity, high acceptance and popularity of ANC in Tanzania. ANC services are popular as a majority of women perceive it as important in monitoring their health and that of the unborn baby. Similar findings were observed in Gambia [40].

The frequency and timing of ANC visits is also important as it could help women to overcome fears of health providers [12]. Due to culture and beliefs, women in many settings do not disclose their pregnancy status and most of them wait until the third trimester. In our study area, early visits by SMPs made it possible for more women to disclose their pregnant status and to book early for ANC. Although not all women were willing to disclose their pregnancy status to SMPs during home visits, as soon as the SMPs had left, a number of women with early pregnancies decided to go to ANC, based on the SMPs general advice and considering that in the following months the "hidden secret" will be known anyhow.

There are limitations to our study. As opposed to other designs with a control group, the pre-post design of the same group is known for its weakness in capturing confounding factors that may affect the study outcome [41]. However, the limitations inherited in the pre-post comparison design were minimized by employing different methods of data collection and data sources. Secondly, the district data do not show any substantial increase during the study period. Although our study results are context specific, the principles and the lessons learned can guide similar interventions in most parts of Tanzania or in other rural settings in Africa.

## Conclusion

Firstly, the study shows that the use of community volunteers in promoting access to obstetric care is feasible and can be effective. Secondly, community level interventions to promote access to obstetric care must be built around functioning health care facilities. Use of community volunteers to follow-up pregnant women can substantially improve utilization of skilled attendant at delivery. For community volunteers approach to be successful, strategies have to target individual women and use other influential people such as men and female gate-keepers and religious leaders. Thirdly, linking community volunteers with health providers is also critical in making a program successful. Finally, through proper training and close follow up, TBAs can become an important pillar in the referral system by referring women with risk factors to higher level of care.

## Competing interests

The authors declare that they have no competing interests.

## Authors' contributions

DM and AJ designed the study. DM participated and supervised the filed work and data collection, data analysis and prepared the manuscript for publication. RM was involved in the study design, data analysis and preparation of the manuscript. AJ offered scientific advice, inputs and critique during the data collection and analysis and throughout the preparation of the manuscript.

All authors read and approved the final manuscript.

## Pre-publication history

The pre-publication history for this paper can be accessed here:

http://www.biomedcentral.com/1471-2393/10/14/prepub

## References

[B1] UNICEF Tracking progress in maternal, newborn & child survival 2008 Geneva: UNICEF

[B2] ICM/FIGO Delivering services and influencing policy: Health care professionals join forces to improve maternal, newborn, and child health Int J Obstet Gynaecol 2009 105 271 274 10.1016/j.ijgo.2009.01.030 19339007

[B3] UN Millennium Declaration 2000 Washington: United Nation

[B4] National Bureau of Statistics Tanzania Demographic and Health Survey 2004-05 2005 Dar-Es-Salaam, Tanzania: National Bureau of Statistics & ORC Macro

[B5] Massawe SN Urassa EN Nystrom L Lindmark G Anemia in women of reproductive age in Dar-es-Salaam, Tanzania. Anemia in women of reproductive age in Dar-es-Salaam, Tanzania East Afr Med J 2002 79 461 466 12625686 10.4314/eamj.v79i9.9117

[B6] Font F Alonso GM Nathan R Lwilla F Kimario J Tanner M Alonso PL Maternal mortality in a rural district of southeastern Tanzania: an application of the sisterhood method Int J Epidemiol 2000 29 107 112 10.1093/ije/29.1.107 10750611

[B7] National Bureau of Statistics {Tanzania} & Macro International Inc. 1997 Tanzania Demographic and Health Survey 1996 1996 Calverton, Maryland: Bureau of Statistics & Macro International

[B8] Urassa E Massawe S Lindmark G Nystrom L Operational factors affecting maternal mortality in Tanzania Health Policy and Planning 1997 12 50 57 10.1093/heapol/12.1.50 10166102

[B9] Moore KM A Behaviour change approach to investigating factors influencing use of skilled care in Home-Bay district, Kenya 2002 Washington, DC: The CHANGE Project/Academy for Education and Development/Manoff Group

[B10] Thaddeus S Maine D Too far to walk: maternal mortality in context Soc Sci Med 1994 38 1091 1110 10.1016/0277-9536(94)90226-7 8042057

[B11] Ross RS Promoting quality maternal and newborn care: A reference manual for program manager 1998 Atlanta: Cooperative for Assistance and Relief Everywhere Inc: CARE

[B12] Kowalewski M Jahn A Kimatta SS Why do at-risk mothers fail to reach referral level? Barriers beyond distance and cost Afr J Reprod Health 2000 4 100 109 10.2307/3583247 11000713

[B13] Rosato M Laverack G Grabman L Tripathy P Nair N Mwansambo C Azad K Morrison J Bhutta Z Perry H Alma-Ata: Rebirth and Revision 5. Community participation: lessons for maternal, newborn, and child health Lancet 2008 372 962 971 10.1016/S0140-6736(08)61406-3 18790319

[B14] Elder J Ayala G Harris S Theories and intervention approaches to health-behavior change in primary care Am J Prev Medicine 1999 17 275 284 10.1016/S0749-3797(99)00094-X 10606196

[B15] Kleinman A Patient and healer in the context of culture 1980 Berkley: University of California Press

[B16] MotherCare Matters Behavioural dimension of maternal and survival. A quarterly newsletter and literature review on maternal and neonatal health 2000 9 3 1 19

[B17] Murray E Mark J A Guide to effective care in pregnancy and childbirth 2000 Oxford: Universiry Press

[B18] Santarelli C Working with individuals, families and community to improve maternal and newborn health 2003 Geneva: (WHO/FCH/RHR/03.11), World Health Organization

[B19] WHO Mother-Baby Package. Implementing Safe Motherhood in developing countries 1994 Geneva: Maternal Health and Safe Motherhood Programme

[B20] Rifkin SB Primary health care, community participation and the urban poor: a review of the problems and solutions Asia-Pacific J Public Health 1987 1 57 63 10.1177/101053958700100211 3452395

[B21] Lankester T Setting up community-based health program: A practical manual for use in developing countries 2000 2 London: McMillan Education Ltd

[B22] Nwakoby B Akpala C Nwagbo D Onah B Okeke V Chukudebelu W Ikeme A Okaro J Egbuciem P Ikeagu A Community contact persons promote utilization of obstetric services, Anambra State, Nigeria. The Enugu PMM Team Int J Obstet Gynaecol 1997 59 219 224 10.1016/S0020-7292(97)00168-9 9389634

[B23] Kiwanuka JS Reducing maternal deaths in Africa 2003 Brazzavile, Republic of Congo: WHO. Regional Office for Africa

[B24] United Nation. The Millennium Development Goals Report 2008 2008 Washington

[B25] Jahn A Kowalewski M Kimatta SS Obstetric care in southern Tanzania: does it reach those in need? Trop Med Int Health 1998 3 926 932 10.1046/j.1365-3156.1998.00323.x 9855407

[B26] Mushi D Mpembeni R Jahn A Knowledge of school children on maternal health and HIV/AIDS. The case of Mtwara, Tanzania BMC Pregnancy and Childbirth 2007 1

[B27] Lennon J Coombs D Application of the LePSA technique to the health education of leprosy patients Leprosy Review 1992 63 145 150 1640782 10.5935/0305-7518.19920019

[B28] Werner D Bower B Helping health worker learn 1982 2 Palo Alto, CA: Hesperian Foundation

[B29] Tanzania Ministry of Health Tanzanian National Reproductive Health and Communication Strategy and Safe Motherhood Initiatives Massages 2001 Dar es Salaam

[B30] District Medical Officer Mtwara District Annual Report 2006

[B31] Essien E Ifenne D Sabitu K Musa A Alti-Mu'azu M Adidu V Golji N Mukaddas M Community loan funds and transport services for obstetric emergencies in northern Nigeria Int J Obstet Gynaecol 1997 59 237 244 10.1016/S0020-7292(97)00171-9 9389637

[B32] Lehmann U Friedman I Sanders D Review of the utilization and effectiveness of community-based health workers in Africa. A Joint learning initiative Human resources for health and development (JLI Working paper 4-1) 2004 Geneva: WHO

[B33] Kendah HB Leigh B Kanu MS Kuteh M Bangura J Seisay A Community motivators promote use of emergency services in Rural Sierra Leon Int J Obstet Gynaecol 1997 59 209 218 10.1016/S0020-7292(97)00167-7 9389633

[B34] Nwakoby BN Use of obstetric services in rural Nigeria J R Soc Health 1994 114 132 136 10.1177/146642409411400304 7932482

[B35] Jessop S Morrissey C Dusch E Cox A Jonas E MotherCare initiatives: Action and results from 31 projects 1993-2000 2000 Arlington, Va.: John Snow Inc

[B36] Rowland S Multiplying light and truth through community health evangelism 2001 Second Udyog Bhavan, Mumbai: GLS Publishing

[B37] Kunene B Beksinska M Involving men in maternity care: Male in maternity project in South Africa 2005 http://www.popcouncil.org/pdfs/frontiers/FR_FinalReports/SA_MIM.pdf

[B38] Mullany BC Becker S Hindin MJ The impact of including husbands in antenatal health education services on maternal health practices in urban Nepal: Results from a randomized controlled trial Health Education Research: Theory and Practice 2006 22 166 176 10.1093/her/cyl060 16855015

[B39] District Medical Officer Mtwara Rural District annual report 2004 2004 Mtwara Rural District

[B40] Walraven G Telfer M Rowley J Ronsmans C Maternal mortality in rural Gambia: levels, causes and contributing factors Bull World Health Organ 2000 78 603 613 10859854 PMC2560770

[B41] Thorogood M Brittan A Coombe Ta Evaluating Interventions-experimental study design in health promotion Evaluating Health promotion Practice and Method 2003 Oxford: Oxford University Press 41 56

